# DeepFRAP: Fast fluorescence recovery after photobleaching data analysis using deep neural networks

**DOI:** 10.1111/jmi.12989

**Published:** 2021-01-16

**Authors:** Victor Wåhlstrand Skärström, Annika Krona, Niklas Lorén, Magnus Röding

**Affiliations:** ^1^ Agriculture and Food, Bioeconomy and Health RISE Research Institutes of Sweden Göteborg Sweden; ^2^ Department of Physics Chalmers University of Technology Göteborg Sweden; ^3^ Department of Mathematical Sciences Chalmers University of Technology and University of Gothenburg Göteborg Sweden

**Keywords:** confocal laser scanning microscopy, deep learning, deep neural network, diffusion, fluorescence recovery after photobleaching, machine learning, regression

## Abstract

Conventional analysis of fluorescence recovery after photobleaching (FRAP) data for diffusion coefficient estimation typically involves fitting an analytical or numerical FRAP model to the recovery curve data using non‐linear least squares. Depending on the model, this can be time consuming, especially for batch analysis of large numbers of data sets and if multiple initial guesses for the parameter vector are used to ensure convergence. In this work, we develop a completely new approach, DeepFRAP, utilizing machine learning for parameter estimation in FRAP. From a numerical FRAP model developed in previous work, we generate a very large set of simulated recovery curve data with realistic noise levels. The data are used for training different deep neural network regression models for prediction of several parameters, most importantly the diffusion coefficient. The neural networks are extremely fast and can estimate the parameters orders of magnitude faster than least squares. The performance of the neural network estimation framework is compared to conventional least squares estimation on simulated data, and found to be strikingly similar. Also, a simple experimental validation is performed, demonstrating excellent agreement between the two methods. We make the data and code used publicly available to facilitate further development of machine learning‐based estimation in FRAP.

**Lay description:**

Fluorescence recovery after photobleaching (FRAP) is one of the most frequently used methods for microscopy‐based diffusion measurements and broadly used in materials science, pharmaceutics, food science and cell biology. In a FRAP experiment, a laser is used to photobleach fluorescent particles in a region. By analysing the recovery of the fluorescence intensity due to the diffusion of still fluorescent particles, the diffusion coefficient and other parameters can be estimated. Typically, a confocal laser scanning microscope (CLSM) is used to image the time evolution of the recovery, and a model is fit using least squares to obtain parameter estimates. In this work, we introduce a new, fast and accurate method for analysis of data from FRAP. The new method is based on using artificial neural networks to predict parameter values, such as the diffusion coefficient, effectively circumventing classical least squares fitting. This leads to a dramatic speed‐up, especially noticeable when analysing large numbers of FRAP data sets, while still producing results in excellent agreement with least squares. Further, the neural network estimates can be used as very good initial guesses for least squares estimation in order to make the least squares optimization convergence much faster than it otherwise would. This provides for obtaining, for example, diffusion coefficients as soon as possible, spending minimal time on data analysis. In this fashion, the proposed method facilitates efficient use of the experimentalist's time which is the main motivation to our approach. The concept is demonstrated on pure diffusion. However, the concept can easily be extended to the diffusion and binding case. The concept is likely to be useful in all application areas of FRAP, including diffusion in cells, gels and solutions.

## 1 INTRODUCTION

Fluorescence recovery after photobleaching (FRAP) is a powerful technique for characterization of different diffusion processes and is used on a regular basis in materials science, pharmaceutics, food science and cell biology.^1^ Diffusive transport processes are indeed important for the understanding of material properties and functionality, in everything from simple viscous liquids to heterogeneous, spatially and temporally fluctuating environments with obstruction effects and interactions with a matrix such as binding effects.^2^ FRAP has been used for estimation of local diffusion coefficients since the 1970s.^3^

In a typical FRAP experiment, fluorescent particles are photobleached by a high‐intensity laser in a bleach region (also referred to as region of interest, ROI) that is typically either circular or rectangular. Unbleached particles will move into the bleach region, leading to a recovery of the fluorescence intensity within the bleach region. Under the assumption that all particles are mobile and that the fraction of bleached particles in the whole sample is negligible, the fluorescence will eventually recover to the pre‐bleach intensity. The rate of recovery is mainly determined by the diffusion coefficient, but also by the size of the bleach region, the amount of bleaching and other parameters. In most FRAP experiments today, a confocal laser scanning microscope (CLSM) is used to image the time evolution of the recovery, using a far lower laser intensity for the imaging than for the bleaching. Quantitative information is obtained by fitting a model for the fluorescence intensity as a function of time, and possibly also of space, to the experimental data.

The case of free diffusion is often generalized to diffusion with binding interactions governed by reaction‐diffusion‐type equations involving on and off binding rate constants, that is, association and disassociation rate constants which are frequently used in cell biology and biomaterials applications.^4^, ^5^, ^6^, ^7^, ^8^, ^9^, ^10^ Other cases involving complex dynamics have also been investigated such as anomalous diffusion,^11^, ^12^ dynamics in phase‐separated protein condensates,^13^ modelling the recovery as a superposition of the recovery by several different physical processes.^14^ Finally, FRAP has been combined with other techniques to gain new insights, for example, fluorescence correlation spectroscopy,^15^, ^16^ selective plane illumination microscopy,^17^ atomic force microscopy^18^ and single‐point illumination for highly localized, single‐molecule FRAP.^19^ However, more complex dynamics beyond free diffusion and integration of FRAP with other techniques is out of the scope of this work.

The physical and mathematical assumptions of the FRAP models and estimation methods as such vary between different approaches, and result in analytical and numerical models of very different complexity. We provide a brief overview of the literature and refer the reader to the review in Ref. 1 for a detailed account. First, many models limit themselves to a circular bleach region and the theoretically sound assumption of a uniform intensity in the bleach region (and outside of it) directly after bleaching. Already at this stage, the assumptions may be violated in complex real‐world samples such as cells; the most common approach is to find a reasonably homogeneous region in a heterogeneous sample and proceed after performing a background subtraction. The average intensity in the bleach region as a function of time after bleaching (i.e. the recovery curve) can be expressed using Bessel functions.^20^, ^21^ If the uniform circular disk is approximated with a Gaussian intensity profile, a closed‐form expression for the full diffusion equation, that is, the spatiotemporal evolution of the fluorescence intensity can be found.^22^ Second, there are multiple common methods for parameter estimation. The conventional estimation paradigm is recovery curve‐based estimation where a model curve is fit to the experimental recovery curve. Less conventionally, some approaches utilize models for the full spatiotemporal solution to the diffusion (or reaction‐diffusion) equation and fit them to the intensity values of all individual pixels in the FRAP image sequence, known as pixel‐based estimation.^22^, ^23^, ^24^, ^25^, ^26^, ^27^ Another approach is Fourier‐domain models that can be useful for anomalous or spatially dependent diffusion.^12^ Third, least squares is used for the most part, implying the assumption of normal distributed noise with constant variance. Some approaches more realistically model the noise as in part proportional to the image intensity and/or Poisson distributed.^21^, ^26^, ^27^, ^28^ Lastly, several important additions to the modelling have been accounted for, such as arbitrary bleach region shapes,^29^ multiple bleach frames,^30^ diffusion during the bleaching phase,^31^ finite bleach resolution (because of a non‐uniform laser beam),^8^, ^21^, ^26^, ^32^ bleaching during imaging^33^ and the raster scan motion of the laser beam during both bleaching and imaging.^9^, ^34^

In previous work, we developed a new numerical model based on spectral methods that incorporates many of the aforementioned features, such as arbitrary bleach region shapes, finite bleach resolution, multiple bleach frames, bleaching during imaging and both recovery curve‐based estimation and pixel‐based estimation.^35^ The downside of many accurate FRAP models including this one is the computational workload. This is for three reasons, namely because of (i) the comparably long execution time of performing a single least squares fit, (ii) the benefit of using multiple initial guesses (up to ten or more) for the parameter vector to ensure convergence to the global optimum when analysing a single dataset and (iii) the fact that it is often useful to make several measurements for determining a single diffusion coefficient in order to robustly assess variability/uncertainty.

In this work, we develop a completely new approach for FRAP analysis based on machine learning for parameter estimation, that we refer to as DeepFRAP. Using the numerical FRAP model developed in Ref. 35, we generate a very large set of simulated recovery curves with realistic noise levels covering a broad range of diffusion coefficients, image intensities, amounts of bleaching and noise levels. We treat parameter estimation as a non‐linear regression problem and use the data for training deep neural network (deep learning) regression models for prediction of the parameters. We implement two different neural network architectures; first, a fully connected network for estimation of all parameters jointly, and second, a set of separate fully connected networks for estimation of individual parameters. The latter is shown to give better performance. The neural networks are extremely fast and can estimate the parameters orders of magnitude faster than least squares. This provides for obtaining, for example, diffusion coefficients as soon as possible, spending minimal time on data analysis. In this fashion, the proposed method facilitates efficient use of the experimentalist's time which is the main motivation to our approach. The performance of the neural network estimation compared to least squares estimation is assessed, and the estimation errors as a function of noise level are strikingly similar. This implies that the neural network estimates can be used as very good initial guesses for the least squares estimation in order to provide a considerable speed‐up for the latter. We also perform an experimental validation in simple solutions which demonstrates excellent agreement between the two methods. We emphasize that although the networks are trained on a particular set of parameters, this work is intended as a proof of concept that can straightforwardly be extended to new cases. Finally, we make the data and code used herein publicly available to encourage and facilitate further development of machine learning‐based estimation in FRAP.^36^

## 2 METHODS

### 2.1 Numerical FRAP model

In a typical FRAP measurement for measuring free diffusion, fluorescent particles are photobleached in a specified bleach region of specified shape. This yields a net flux of unbleached particles into the bleach region at a rate governed by the diffusion coefficient. In turn, this leads to recovery of the fluorescence intensity in the bleach region through the time evolution of the concentration of the (still) fluorescent particles. In Ref. 35, we developed a detailed numerical model for the spatiotemporal concentration profile c(x,y,t) of fluorescent particles. If the bleach region is sufficiently extended in the axial dimension, for example, by means of a small numerical aperture, it can be approximated by an infinite cylinder elongated in the axial dimension. Thus, it follows that there is negligible net diffusion in the z direction, that is, orthogonal to the focal plane. Making this approximation is a common practice and it implies that only two‐dimensional motion has to be considered, although the validity of the approximation varies with samples and experimental settings.^1^ Let the concentration initially be $c\left(x,y\right)=c_{0}$ everywhere. Directly after the first bleach frame, the concentration profile is

(1) $$c\left(x,y\right)=\left\{\begin{array}{cc} c_{0}\alpha , & \left(x,y\right)\in \Omega \\ c_{0}, & \left(x,y\right)\notin \Omega \end{array}\right.,$$

where α is a bleaching parameter and Ω is the bleach region, centred in $\left(x_{c},y_{c}\right)$. Note that in contrast to many other models, the bleaching parameter is interpreted as the relative decrease in intensity due to bleaching rather than the amount of bleaching. The bleach region can have any shape but is typically either circular with radius r or rectangular with dimensions $l_{x}$ and $l_{y}$. If more than one bleach frame is used, expressing the concentration profile directly after bleaching in general requires numerical computation and cannot be easily stated here, but the principle is similar. Also, Equation (1) is never observed due to the fact that the first postbleach frame is acquired with some delay after the bleaching. The evolution of the concentration c(x,y,t) is described by the standard diffusion equation

(2) $$\frac{\partial c}{\partial t}=D\nabla^{2}c,$$

for a diffusion coefficient D. We develop a numerical solver based on spectral methods to generate simulated FRAP pre‐ and post‐bleach images of size N×N pixels (N=256 throughout this work). We solve the diffusion equation in a two‐dimensional domain with periodic boundary conditions and size (N+2M)×(N+2M) grid, where M is the padding (we use M=128). Time stepping is performed in the Fourier domain, and bleaching is performed in the spatial domain. The numerical solution can be computed for arbitrary numbers of prebleach frames $n_{\mathrm{prebleach}}$, bleach frames $n_{\mathrm{bleach}}$ and postbleach frames $n_{\mathrm{postbleach}}$, with time lag Δt between consecutive frames. Bleaching is represented by element‐wise multiplication of the concentration with an (N+2M)×(N+2M) matrix which is 1 outside the bleach region and α inside. Time stepping is performed in the following fashion. The concentration c(x,y,t), with t corresponding to an arbitrary pre‐bleach, bleach or post‐bleach frame, is transformed to its spectral representation $\hat{c}\left(\xi ,\eta ,t\right)$ using the two‐dimensional Fast Fourier Transform (FFT). In the spectral domain, the single PDE in Equation (2) becomes ${\left(N+2M\right)}^{2}$ independent ODEs of the form

(3) $$\frac{\partial \hat{c}\left(\xi ,\eta ,t\right)}{\partial t}=-\left(\xi^{2}+\eta^{2}\right)D\hat{c}\left(\xi ,\eta ,t\right)$$

for each grid point (ξ,η). A closed‐form solution is available for any time (step), and because we always want to make a jump Δt in time, it takes the form

(4) $$\hat{c}\left(\xi ,\eta ,t+\Delta t\right)=e^{-\left(\xi^{2}+\eta^{2}\right)D\Delta t}\hat{c}\left(\xi ,\eta ,t\right).$$

After time‐stepping, we use inverse FFT to obtain c(x,y,t+Δt). The numerical solver is implemented in MATLAB (The Mathworks, Natick, MA). More features are implemented in the solver than mentioned here but are not used in the present work, that is, accounting for an immobile fraction of particles, bleaching during imaging, finite bleach and finite imaging resolutions and reaction–diffusion models for diffusion with binding.

**FIGURE 1:**
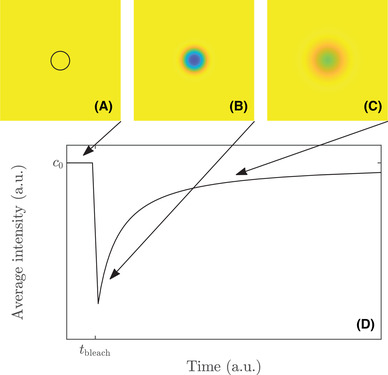
An illustration of the FRAP experiment, using simulated data from the model used herein, showing (A) a pre‐bleach frame with a circular bleach region indicated (black ring), (B and C) two different post‐bleach frames, and (D) the recovery curve, computed as the average intensity within the bleach region

It is a standard assumption in FRAP that the sample and the acquisition parameters are such that the fluorescence intensity is proportional to the concentration. Because of that and of linearity of Equation (2), particle concentration and fluorescence/image intensity can be used interchangeably although they are not the same. The notations c and $c_{0}$ will be used to denote dimensionless fluorescence intensities, corresponding to rescaled concentrations. In Figure 1, we illustrate the FRAP experiment with simulated FRAP data from the model.

### 2.2 Noise models

The most common assumption in FRAP is that the experimental noise is normal distributed and independent between pixels, that it has zero mean, and that the variance $\sigma^{2}\left(c\left(x,y,t\right)\right)$ for a concentration c(x,y,t) is generally of the form

(5) $$\sigma^{2}\left(c\left(x,y,t\right)\right)=a+a^{\prime }c\left(x,y,t\right),$$

where a represents constant noise and $a^{\prime }$ represents noise proportional to the mean intensity (reflecting the underlying Poisson nature of the photon counts).^21^, ^26^, ^27^, ^28^ If $a^{\prime }>0$, it would be preferable to estimate a and $a^{\prime }$ from independent calibration data from a homogeneous fluorescent solution, with varying laser intensities, otherwise using settings identical to those for the FRAP experiment.^27^ We assume, as is mostly done, that $a^{\prime }=0$ and that the assumption of constant noise is sufficiently accurate; in particular if the amount of bleaching is not too large, that is, if α is not too small.

### 2.3 Conventional parameter estimation

As mentioned above, it is possible to fit a model for c(x,y,t) using the intensity values of all individual pixels in the FRAP image sequence (pixel‐based estimation). In this work, we are only concerned with the simpler and faster recovery curve‐based estimation, which is the conventional and most frequently used approach. Further, training neural networks on recovery curve data is a much simpler task than training on the full image data (especially considering the high dimensionality of image sequence/video data). First, from a measurement we obtain the concentration profile $c_{\exp}\left(x,y,t\right)$ and compute the experimental recovery curve $F_{\exp}\left(t\right)$ by

(6) $$F_{\exp}\left(t\right)=\sum_{x,y}m\left(x,y\right)c_{\exp}\left(x,y,t\right).$$

Here, m(x,y) is a normalized indicator function (matrix) of the bleach region, such that Equation (6) produces the average intensity inside the bleach region (because of the finite resolution of the computational grid, the edges of the bleach region are somewhat smoothed as represented by the indicator function). The model recovery curve F(t) is computed analogously. The parameters are estimated by nonlinear least squares,

(7) $$\hat{\theta }=\min_{\theta }{\left(F_{\exp}\left(t\right)-F\left(t\right)\right)}^{2},$$

where $\hat{\theta }$ is the value of the parameter vector θ that corresponds to the global minimum of the sum of squares on the right‐hand side (we suppress that F(t) depends on θ in the notation). In the most general free diffusion case covered by the model in Ref. 35, θ will contain the diffusion coefficient D, the mobile fraction, the initial concentration $c_{0}$, the bleaching parameter α, imaging bleach parameter and a bleach and imaging resolution parameter. In practice, several of these are eliminated by the experimental design (e.g. by ensuring that laser levels are sufficiently small as to avoid imaging bleach). In this work, we are only concerned with $\theta =(D,c_{0},\alpha )$. Contrary to some other FRAP models, both pre‐ and post‐bleach data are used for estimation. The estimation procedure is implemented in MATLAB (The Mathworks) using lsqnonlin.

### 2.4 Non‐linear regression and neural networks

The conventional least squares fitting above is essentially a type of curve fitting or nonlinear regression. The problem of estimating the parameters can be viewed as nonlinear regression in another sense, namely, as a supervised learning problem in machine learning: Given a set of inputs x (recovery curves) and the corresponding set of outputs or targets y (parameter vectors of the FRAP model), the aim is to learn a representation of the mapping y=f(x). Assuming that N samples are available, the classical approach to regression in this setting is to model the i:th target as

(8) $$y_{i}=f\left(x_{i};w\right)+\varepsilon_{i},$$

where f belongs to some class of mappings with parameters w. Further, $\varepsilon_{i}$ is normal distributed, zero‐mean noise, naturally implying that we should minimize a (weighted) mean squared error (MSE) loss,

(9) $$\mathrm{MSE}\left(x,y;w\right)=\frac{1}{3N}\sum_{i=1}^{N}\zeta_{i}{\left|y_{i}-f\left(x_{i};w\right)\right|}^{2},$$

for all parameters jointly, where the factor 1/3 comes from the fact that we herein will consider target vectors of 3 parameters. For the jth parameter individually, the same loss becomes

(10) $$\mathrm{MSE}\left(x,y^{\left(j\right)};w\right)=\frac{1}{N}\sum_{i=1}^{N}\zeta_{i}{\left(y_{i}^{\left(j\right)}-f{\left(x_{i};w\right)}^{\left(j\right)}\right)}^{2}.$$

Here, $\zeta_{i}$ is a weight that depends on $\varepsilon_{i}$. With MSE (from here on, with MSE we mean weighted MSE), the regression effectively becomes non‐linear least squares as in the conventional estimation, but with respect to the parameter vector of the FRAP model rather than the recovery curves themselves. Many different regression models can be used to find a good approximation for y=f(x). Artificial neural networks (ANNs) that we use in this work are one of the most versatile paradigms for representing highly non‐linear functions in supervised learning, whether the ultimate goal is regression or classification.

One of the fundamental ANNs is the perceptron,^37^ where the function representation f is chosen such that

(11) $$y=f\left(x\right)=g\left(w^{T}x+b\right),$$

where w are the representation weights, b is called the bias or threshold and g a non‐linear activation function. If g is chosen as a linear function, this would constitute ordinary linear regression. A multilayer perceptron (MLP) or fully connected neural network, is a chain of multiple layers of maps as defined in Equation (11). An MLP with n layers may be defined iteratively as

(12) $$\begin{array}{cc} y= & \;g_{n}\left(h_{n}\right), \end{array}$$

(13) $$\begin{array}{cc} h_{\ell }= & \;w_{\ell }^{T}g_{\ell -1}\left(h_{\ell -1}\right)+b_{\ell },\;\ell =2,\ldots ,n-1, \end{array}$$

(14) $$\begin{array}{cc} h_{1}= & \;w_{1}^{T}x+b_{1}, \end{array}$$

where $h_{\ell }$ is known as the hidden state of layer ℓ, ℓ=1,…,n. The final activation function $g_{n}$ is usually the identity function in regression problems. The combination of a non‐linear activation function and multiple network layers grants neural networks the theoretical capacity to approximate arbitrarily complex functions, sometimes known as the universal approximation theorem.^38^, ^39^

Finding the optimal function representation in terms of the parameters $w_{\ell },b_{\ell },\;\ell =1,\ldots ,n$ is usually done by variations of the stochastic gradient descent (SGD) algorithm for optimization and backpropagation.^40^ Although the choice of activation function is not key to the theoretical approximation capabilities of a network, historically popular choices such as the hyperbolic tangent and other sigmoid activations may lead to saturation of the gradient and thus lowered performance of the optimizer. State‐of‐the‐art methods conventionally use a variation of the rectified linear unit (ReLU),

(15) $$\text{ReLU}\left(x\right)=\max\left(x,0\right)=\left\{\begin{array}{cc} x, & x>0\\ 0, & x\leq 0 \end{array}\right.,$$

which is non‐differentiable at the origin, but leads to less saturation.^41^

## 3 RESULTS AND DISCUSSION

For both simulated and experimental data, we limit the investigation to settings that accommodate many normal experimental use‐cases in which we perform FRAP, which typically involve free diffusion in liquids or diffusion in gels. We stress that the principles are equally valid in any other setting in which high‐quality FRAP measurements can be performed, such as in cells, which would require smaller fields of view and bleach regions. As we shall elaborate on below, the neural network‐based estimation can be adapted to new settings in a straightforward manner. We use a resolution of N×N = 256×256 pixels, pixel size Δx=0.7598 µm (providing a field of view of 194.5×194.5 µm), and time lag Δt=0.265 s between consecutive frames. Further, we use a circular bleach region with 30 µm diameter, centred in the field of view (also, disregarding a finite bleach resolution since the impact on such a large bleach region would be insignificant). We use $n_{\mathrm{prebleach}}=10$ pre‐bleach frames, $n_{\mathrm{bleach}}=4$ bleach frames and $n_{\mathrm{postbleach}}=100$ post‐bleach frames. We assume that there is no bleaching during imaging and that the mobile fraction is 1, that is, that there are no immobile particles. Further we assume that the system is homogeneous and isotropic and hence that the initial concentration before bleaching is a constant $c_{0}$. Finally, we use the common assumption that the noise variance is constant, that is, no intensity‐proportional noise.

**FIGURE 2:**
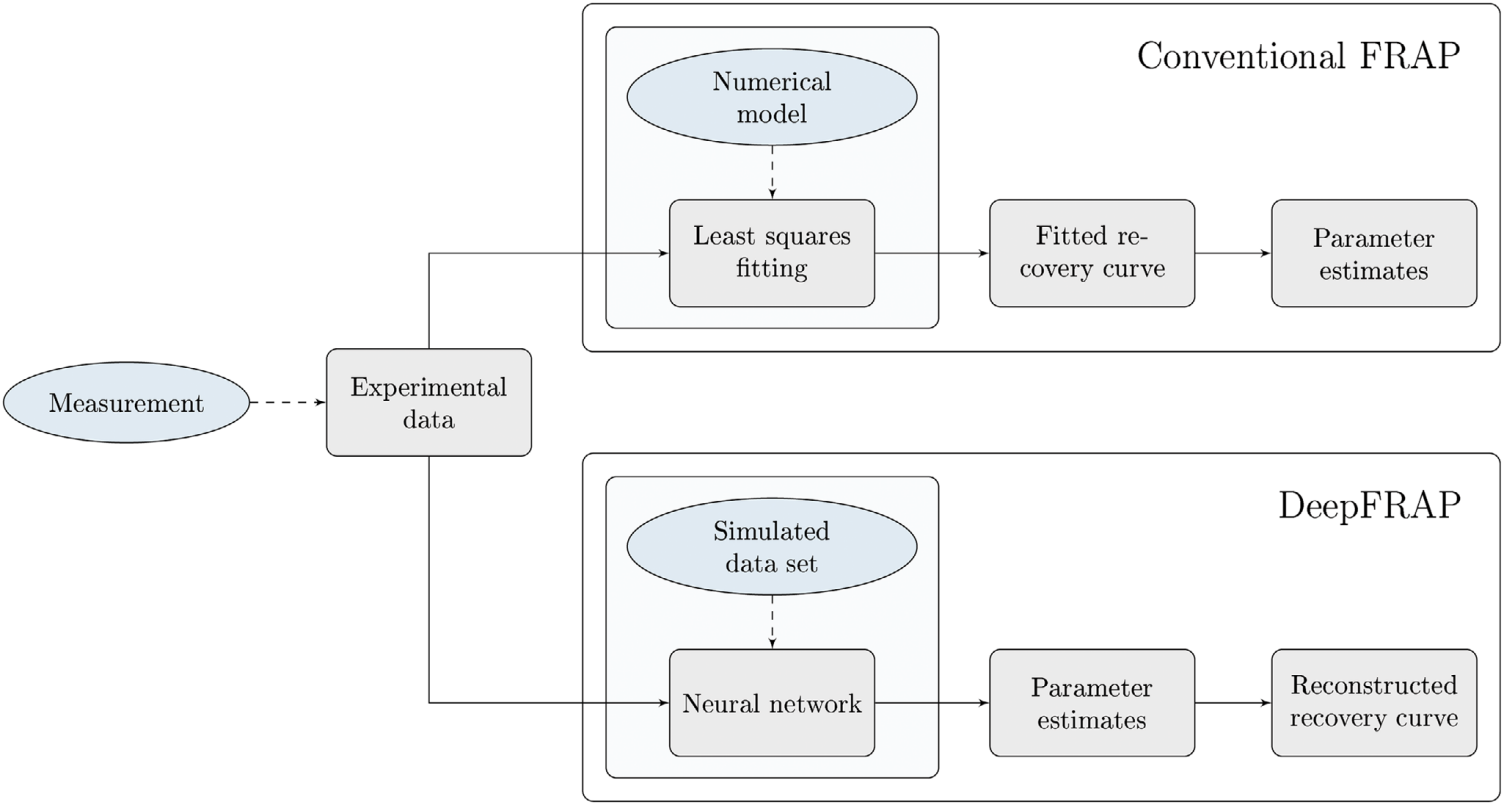
Schematic overview of the estimation workflow to illustrate the differences between conventional FRAP and our DeepFRAP approach

To illustrate the differences between conventional FRAP and our DeepFRAP approach, we give a schematic overview in Figure 2. After performing a measurement, we obtain experimental data, typically through some preprocessing steps (like background subtraction, and computation of the recovery curve from the image data). Then, in the conventional FRAP, we feed the experimental data to a non‐linear least squares optimizer, that uses the numerical FRAP model to find the model recovery curve that is closest to the experimental recovery curve, in terms of the sum of squared residuals between the two. From the fitted recovery curve, parameter estimates are obtained. In contrast, in the proposed DeepFRAP approach, a neural network is trained using simulated data from the numerical FRAP model with realistic noise levels, typically prior to the measurement. The neural network is trained to predict parameters directly rather than finding the model recovery curve closest to the experimental recovery curve. A model recovery curve can then be reconstructed from these parameters, but it is not explicitly part of any fitting. For both methods, the noise variance a is estimated afterwards using the residuals between the experimental and model recovery curves. We elaborate on the DeepFRAP approach in the subsections below.

### 3.1 Data generation

We generate a training set for training the neural networks, a validation set for hyperparameter optimization of the neural networks, and finally a test set for final assessment of performance. Each data set consists of a large set of random input vectors (recovery curves) and random output vectors (parameter values) representative of the parameter domain in which the neural networks will be trained. The dataset sizes are $2^{20}$ (1,048,576) for the training set and $2^{18}$ (262,144) each for the validation and test sets. Data are generated using the settings above and for random values for each of the parameters of interest, which are the diffusion coefficient D, the initial concentration $c_{0}$, the bleach parameter α and the noise variance a. In Table 1, the distributions for random sampling of parameter values are given.The interval of diffusion coefficients covers a range of biologically relevant molecules such as short DNA molecules ($∼10^{-12}$ m${}^{2}$/s),^42^ bovine serum albumin ($∼6\times 10^{-11}$ m${}^{2}$/s),^43^ β‐lactoglobulin ($∼10^{-10}$ m${}^{2}$/s)^44^ and sodium flourescein ($∼4\times 10^{-10}$ m${}^{2}$/s).^45^ The reason why D is chosen log‐uniformly distributed (i.e. $\log_{10}D$ is uniformly distributed in [−12,−9]) is that considering the broad range of values over three orders of magnitude, a log‐uniform distribution provides a good means of prioritizing small and large diffusion coefficients equally in the data set (otherwise, ∼ 90% of the randomly picked diffusion coefficients will be larger than $10^{-10}$ m${}^{2}$/s). We argue similarly for the noise variance a, where the range is picked approximately centred around typical experimental values in log scale. The range for $c_{0}$ is motivated by the fact that it is desirable to be close to 1 for an optimal signal‐to‐noise ratio, but not too close as to avoid saturation in any pixels. (It would be possible to normalize all data with respect to the average prebleach intensity to get, say, $c_{0}=1$, thereby avoiding to estimate $c_{0}$. However, the post‐bleach intensities also contain information about $c_{0}$ which would then be lost, and given that $c_{0}$ is by far the simplest parameter to estimate, including it has little impact on the accuracy of other estimates.) The range of α is motivated from both a data standpoint and a physics standpoint: The value should not be too close to 1 to ensure good contrast in the post‐bleach images, and not too small as to avoid nonlinearities in the bleaching. Internally in the numerical code, the diffusion coefficient is specified in units of pixels${}^{2}$/s, avoiding the SI units for numerical reasons. In the neural network workflow, we similarly use $D^{★}=\log_{10}D$ (for D in units of pixels${}^{2}$/s). The distribution of $D^{★}$ is uniform in ∼[0.2386,3.2386], hence with a range in the same order as for $c_{0}$ and α.

**TABLE 1:** Distributions of parameter values in the generated data

Parameter	Distribution
D	Log‐uniform in $\left[10^{-12},10^{-9}\right]$ m${}^{2}$/s
$c_{0}$	Uniform in [0.5,1]
α	Uniform in [0.45,0.95]
a	Log‐uniform in $\left[10^{-4},10^{-2}\right]$

**FIGURE 3:**
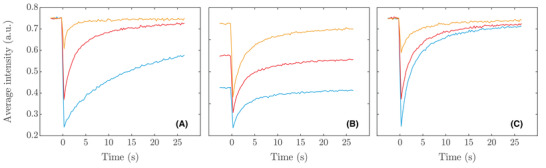
Examples of simulated recovery curves from the training data for different parameters, varying one parameter at a time. In (A), recovery curves for $c_{0}=0.75$, α=0.70 and D values $5\times 10^{-12}$ m${}^{2}$/s (blue), $5\times 10^{-11}$ m${}^{2}$/s (red) and $5\times 10^{-10}$ m${}^{2}$/s (yellow) are shown. In (B), recovery curves for $D=5\times 10^{-11}$ m${}^{2}$/s, α=0.70 and $c_{0}$ values 0.5 (blue), 0.7 (red) and 0.9 (yellow) are shown. In (C), recovery curves for $D=5\times 10^{-11}$ m${}^{2}$/s, $c_{0}=0.75$ and α values 0.5 (blue), 0.7 (red) and 0.9 (yellow) are shown

For each sample in the datasets, FRAP image data is simulated using the numerical algorithm presented in Methods. Normal distributed random noise with variance a is added to the data, and the recovery curve is extracted from the image sequence by computing the average intensity in the bleach region for each point in time as described in Equation (6). For the ith sample, the resulting data is a 110‐dimensional vector $x_{i}$ (merging pre‐ and post‐bleach data), which constitutes one sample of the input data for the neural networks. The target data are the three‐dimensional parameter vector $y_{i}=\left(D_{i}^{★},c_{0,i},\alpha_{i}\right)$ (analogous to least squares, $a_{i}$ can be computed after estimation using the experimental recovery curve and the fitted model). In Figure 3, we show some examples of the generated recovery curves. As can be seen, there is a very broad distribution of behaviour among the shown curves. For example, some curves are not even close to recovering to their prebleach intensity within the experimental time, but this is a deliberate choice in order to cover the range of diffusion coefficients that are reasonable to estimate using the chosen experimental parameters with a generous margin. It is worth pointing out that this does not imply that the neural networks will be able to learn to utilize data from recovery curves with incomplete recovery better than least squares, but including the data will improve the performance of the network.

The benefit of using simulated data for a machine learning task is that because the simulation mechanism is the same, the data for training, validation, and test will be identically distributed. Further, because different random seeds are used, the datasets will be independent. Hence data leakage, that is, overestimated performance as a result of the optimizer having access to the validation set during training, is avoided.

### 3.2 Neural networks

We optimize neural networks with respect to MSE loss as per Equations (9) and (10). In the first attempts, we did not introduce any weights $\zeta_{i}$ in the loss functions; the consequence was that the loss function prioritized errors for small and large noise variances equally, as a consequence focusing primarily on the errors for large noise variances because they effectively become outliers. A better approach is to use the fact that the variance of parameter estimates in conventional estimation, like least squares (or maximum likelihood), are approximately linearly proportional to the variance, or noise, in the data. Because the MSE is related to the variance of the parameter estimates, it makes sense to select the weights $\zeta_{i}$ such that $\zeta_{i}\propto 1/a_{i}$ (and normalized such that the sum of all weights in each dataset is 1). This drastically improves estimation performance of the neural networks for lower noise variances, and the possibility of introducing such a weighting is the main reason for working with the assumption of constant normal distributed noise.

Before training of neural networks, we perform least squares estimation on $2^{16}$ (65,536) samples generated from the same distribution of parameters as for the training data, and compute the MSE in the same manner. This provides benchmark values that are useful for assessing the performance of the neural networks. In Table 2, the MSE for all parameters jointly and for the individual parameters are shown for least squares estimation (note that the factor 1/3 in Equation (9) is the reason why the joint MSE is smaller than the largest individual MSE).These losses give an approximate indication of the lowest attainable loss for any neural network. As we shall see, the MSE losses for least squares do not constitute sharp lower bounds. Of course, the noticeable differences in MSE for the individual parameters (several orders of magnitude) reflect the difficulty of estimating the different parameters (the impact of having non‐standardized parameter ranges in Table 1 is found to be negligible in comparison; hence we do not introduce standardized parameter ranges). The difficulty of estimation essentially reflects the information content of the recovery curve which boils down to the Fisher information of the corresponding likelihood function.

**TABLE 2:** MSE losses for least squares estimation, showing results for all parameters jointly and individually

Parameter	MSE
All	$4.0011\times 10^{-5}$
$D^{★}$	$1.0429\times 10^{-4}$
$c_{0}$	$2.0511\times 10^{-8}$
α	$1.5723\times 10^{-5}$

**FIGURE 4:**
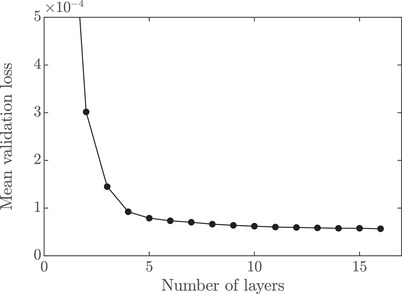
Minimum validation loss over 200 epochs, averaged over 20 runs for each number of layers, as a function of the number of layers

The neural networks are implemented in Tensorflow 2.1.0.^46^ The conventional SGD is selected as the optimizer (found to perform better in this case than the Adam optimizer^47^). In the first step, we consider using fully connected neural network architectures for estimation of all three parameters jointly. We perform hyperparameter optimization with respect to the optimizer and the network architecture. For the optimizer, we vary batch size, learning rate and momentum. For the network, we vary the number of layers, the number of nodes per layer, and the activation function. We use batch size 1024 and momentum 0.99. Further, we find that the exponential linear unit (ELU) activation,^48^

(16) $$\mathrm{ELU}\left(x\right)=\left\{\begin{array}{cc} x, & x>0\\ \gamma \left(\exp(x)-1\right), & x\leq 0 \end{array}\right.,$$

with γ=1, yields slightly better results than the more typical ReLU or tanh activations. We investigate the influence of the number of nodes per layer and the number of layers. We find that there is no discernible gain with more than 128 nodes per layer. As for the number of layers, we investigate using 1 to 16 layers with 128 nodes per layer. This investigation is performed with a constant learning rate of $10^{-3}$. In Figure 4 we show the obtained minimum validation loss when training for 200 epochs (iterations over the whole data set), averaged over 20 runs for each number of layers. Although the validation loss continues to decrease slightly even for very deep networks, the returns of adding more layers are rapidly diminishing. Given that the execution time for a single epoch of training is approximately linearly proportional to the number of layers, eight layers is selected as a reasonable trade‐off. The final learning rate schedule is optimized after fixing all other hyperparameters, using an initial increase followed by a step‐wise decay: First, for 50 epochs, the learning rate η is linearly increased from $10^{-3}$ to $10^{-1}$. Learning rates higher than $10^{-1}$ otherwise resulted in a diverging loss. Second, we use a step‐wise decay, where the learning rate is decreased in a log‐linear fashion such that $\log_{10}\eta$ is −1,−1.25,−1.5,..., with 8000 epochs for each value. To account for seed sensitivity, that is, the dependence on the random initialization of the network and random shuffling of data, four independent networks are trained, using Glorot (uniform) weight initialization. The training is performed on a dual AMD Epyc 7542 with 128 threads, and is run for 7 days (168 h), after which slightly more than the first 6 of the 8,000 epoch sections were done (48,000 epochs). The network yielding the lowest validation loss for any epoch is selected for testing. In Table 3, the results for training, validation, and test sets are shown, both for the MSE for all parameters and for the individual parameters.Importantly, the MSE losses for least squares do not constitute sharp lower bounds as can be seen by comparing Tables 3 and 2. Indeed, the joint test MSE and the individual test MSE for $D^{★}$ are both lower for the neural network than for least squares. The reason why this is possible is that least squares estimation does not optimize the fit with respect to this loss and not even with respect to the parameter values per se; it optimizes the fit with respect to the sum of squared differences between the recovery curve data and the recovery curve model, see the schematic overview in Figure 2. In contrast, in a comparison between least squares and neural networks with respect to the sum of squared residuals for the recovery curve, least squares would be superior by definition (provided the global optimum is found which is not guaranteed in non‐linear least squares). For $c_{0}$ and α, the networks are not able to surpass least squares in terms of MSE, although the existence of a neural network representation that can is guaranteed as per the universal approximation theorem. Albeit, finding the correct weights of this neural network is non‐trivial.

**TABLE 3:** MSE losses for the best‐performing network for prediction of all three parameters jointly, showing results for all parameters jointly and individually for the training, validation and test sets

	MSE		
Parameter	Train	Val	Test
All	$3.6782\times 10^{-5}$	$3.7809\times 10^{-5}$	$3.8842\times 10^{-5}$
$D^{★}$	$9.4660\times 10^{-5}$	$9.7368\times 10^{-5}$	$1.0038\times 10^{-4}$
$c_{0}$	$6.3904\times 10^{-8}$	$6.4715\times 10^{-8}$	$6.4995\times 10^{-8}$
α	$1.5623\times 10^{-5}$	$1.5994\times 10^{-5}$	$1.6081\times 10^{-5}$

**FIGURE 5:**
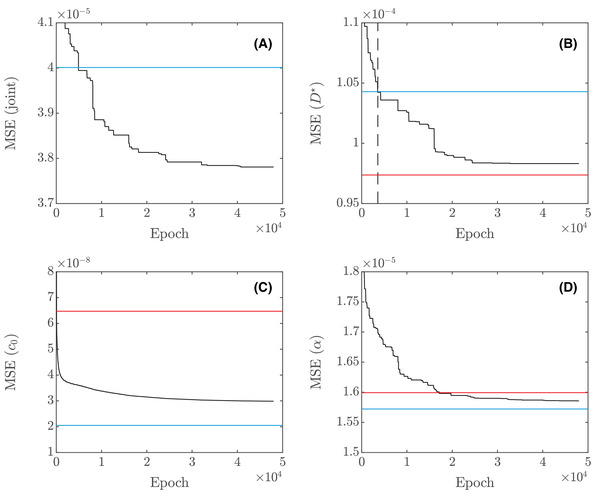
Loss curves (black) for (A) the joint network, (B) the $D^{★}$ network, (C) the $c_{0}$ network and (D) the α network, showing the cumulative minimum of the validation loss rather than the loss itself. In all four cases (A)–(D), the least squares benchmark value for the loss is shown (blue). Further, for the three networks for individual parameters in (B)–(D), the corresponding loss for the joint network but for each individual parameter is shown (red). In (B), the point where the obtained loss becomes lower than the least squares benchmark value for the loss is shown (vertical, dashed, black line)

That the neural network yields better results specifically for the parameter for which the individual loss is largest is no surprise. Indeed, optimization with respect to the MSE is bound to prioritize reducing the largest prediction errors. Also, with the chosen network architecture which has 130,179 weights, 129,792 of them are actually shared between the three parameters (all weights except for the final layer). Hence, there will unavoidably be a trade‐off between the loss of the different parameters. This introduces an implicit weighting between the parameters for which there is no direct counterpart in least squares and, inevitably, D will be prioritized in the current setting. One manner in which to further approach the least squares estimation would be to introduce different weights for the parameters in the MSE to account for the vastly different magnitudes of the individual losses. Instead, we suggest to train separate networks for the three individual parameters, using an architecture identical to the original network except for the final layer, where two output values are removed in each network. These networks are trained using the same settings and training time as above. Once again, to account for seed sensitivity, four independent networks (for each parameter) are trained. The training of all (16) networks are executed in parallel. The three identified optimal networks are then merged into a single neural network for faster execution when calling TensorFlow. This merged network, which is not a fully connected network but rather constitutes three fully connected blocks executed in parallel, has 389,763 weights, and all weights are optimized with respect to the single parameter scalar output only. In Table 4, the results for training, validation and test sets are shown, both for the MSE for all parameters and for the individual parameters.Separate training with respect to the different parameters removes the implicit weighting of the parameters, and indeed, the losses for $c_{0}$ and α have decreased, and the relative magnitudes of the losses match least squares estimation somewhat better. In Figure 5, the validation loss curves are shown for the best of the four types of networks trained. We actually show the cumulative minimum of the validation loss rather than the loss itself, because the latter becomes hard to visualize when the number of epochs is very large. One noticeable feature of the loss curve for $D^{★}$ is that already after approximately 4000 epochs (approximately 12 h), the network performs better than least squares. This indicates that reasonable results should be attainable with far less training time. Interestingly, the loss for $D^{★}$ has increased although this new network should have somewhat larger information capacity with regard to all parameters, including $D^{★}$. We noticed this behaviour for all optimizer settings studied, and there are at least three possible explanations: (i) seed sensitivity, although such a consistent behaviour is probably not caused by this alone, (ii) the weights of the joint network are already focused on prediction of $D^{★}$ to a large extent anyway and (iii) the loss landscape will be completely different (in terms of the location of local minima) for the joint network and the separate $D^{★}$ network, which may somehow lead to easier convergence of the joint network with respect to estimation of $D^{★}$. Nevertheless, the true explanation remains elusive.

**TABLE 4:** MSE losses for the best‐performing network for prediction of all three parameters separately, showing results for all parameters jointly and individually for the training, validation and test sets

	MSE		
Parameter	Train	Val	Test
All	$3.6496\times 10^{-5}$	$3.8068\times 10^{-5}$	$3.9192\times 10^{-5}$
$D^{★}$	$9.3943\times 10^{-5}$	$9.8320\times 10^{-5}$	$1.0160\times 10^{-4}$
$c_{0}$	$2.9811\times 10^{-8}$	$2.9829\times 10^{-8}$	$2.9864\times 10^{-8}$
α	$1.5517\times 10^{-5}$	$1.5854\times 10^{-5}$	$1.5950\times 10^{-5}$

Although none of the differences in the results between Table 3 and Table 4 are crucial for any practical purposes, they illustrate the interesting point that the combined loss does not prioritize the parameters equally. In the rest of this work, we use the (parts of) networks that provide the best performance for each parameter separately, i.e. we extract the $D^{★}$ part from the joint network and merge it with the separate $c_{0}$ and α networks to obtain a single final network.

### 3.3 Comparison of estimation methods

**FIGURE 6:**
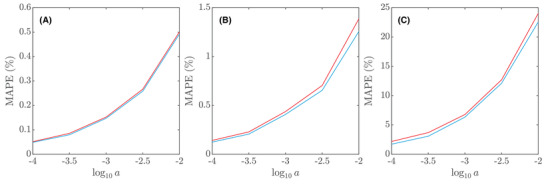
MAPE error for D and for least squares (blue) and the neural network (red) as functions of the noise variance a, showing (A) $D=10^{-11.5}$ m${}^{2}$/s and α=0.5, (B) $D=10^{-10.5}$ m${}^{2}$/s and α=0.7 and (C) $D=10^{-9.5}$ m${}^{2}$/s and α=0.9

We perform a comparison of least squares and the neural network by generating large numbers of simulated data sets for each of a number of distinct parameter values. Using the same parameters as above otherwise, we generate 256 recovery curves for each of the D values $\left\{10^{-11.5},10^{-11},10^{-10.5},10^{-10},10^{-9.5}\right\}$ m${}^{2}$/s (we return to linear scale and SI units from here on), for $c_{0}=0.75$ throughout, for the α values {0.50,0.60,0.70,0.80,0.90}, and for the a values $\left\{10^{-4},10^{-3.5},10^{-3},10^{-2.5},10^{-2}\right\}$. Let ${\hat{D}}^{\left(\mathrm{ls}\right)}$ and ${\hat{D}}^{\left(\mathrm{nn}\right)}$ be the estimates for D from the least squares and the neural network, with similar notation for the other parameters. The estimation methods are compared in terms of mean absolute percentage error (MAPE). For example, for D and for the neural network, MAPE is computed as

(17) $$\mathrm{MAPE}\left({\hat{D}}^{\left(\mathrm{nn}\right)}\right)=\frac{1}{N}\sum_{i}\left|\frac{{\hat{D}}_{i}^{\left(\mathrm{nn}\right)}-D}{D}\right|\times 100\%,$$

for some combination of true values of D, α and a. In Figure 6, a comparison of MAPE errors for ${\hat{D}}^{\left(\mathrm{ls}\right)}$ and ${\hat{D}}^{\left(\mathrm{nn}\right)}$ as a function of a and for three different combinations of D and α values is shown. The general appearance is representative of all 25 combinations, although the magnitudes differ. The MAPEs for ${\hat{D}}^{\left(\mathrm{ls}\right)}$ are in the range 0.05%–22.57% and the MAPEs for ${\hat{D}}^{\left(\mathrm{nn}\right)}$ are in the range 0.05%–24.02%. They also follow each other rather closely (ρ≈0.999), further indicating that the two methods use the information in the recovery curves almost equally well. The MAPEs for ${\hat{\alpha }}^{\left(\mathrm{ls}\right)}$ are in the range 0.005%–5.07% and the MAPEs for ${\hat{\alpha }}^{\left(\mathrm{nn}\right)}$ are in the range 0.006%–4.41%. Again, they follow each other rather closely (ρ≈0.997). Interestingly, for 4 of the 125 parameter combinations, the neural network obtained a smaller MAPE for D than least squares, and similarly for 7 out of 125 for α. However, in the few cases where the neural network performs somewhat better, the difference is very small. The neural network inherits all the limitations of the numerical model and, as stated before, by definition cannot be better than least squares in terms of the residual sum of squares. Nevertheless, it could in principle be better in terms of MAPE (in some parts of the parameter space). That the data indicate this may actually be coincidental though, due to, for example, a finite data set size. With that in mind, we do not claim that the neural network performs better anywhere in the parameter space, but rather that its performance is strikingly similar to least squares. For $c_{0}$, all MAPEs are below 0.11%. Estimation of neither $c_{0}$ nor other parameters is very dependent on the true value of $c_{0}$, therefore sticking to $c_{0}=0.75$ provides a representative picture.

### 3.4 Evaluation on experimental data

To illustrate the performance of the method on real data, we perform an experimental validation on two samples of 100 ppm (0.01 w/w%) sodium fluorescein salt (Sigma‐Aldrich, St. Louis, MO) sucrose‐water solutions. In water, the diffusion coefficient of sodium fluorescein at ambient conditions is approximately $4\times 10^{-10}$ m${}^{2}$/s.^35^, ^45^ ^49^, ^50^ The sodium fluorescein is dissolved in sucrose‐water solutions with 32 w/w% sucrose and 56 w/w% sucrose. In this manner, we obtain samples with two different diffusion coefficients that are approximately $10^{-10}$ m${}^{2}$/s and $10^{-11}$ m${}^{2}$/s at ambient conditions.

The two samples are prepared by placing 7 µl of the solutions in SecureSeal spacers with thickness 120 µm (Grace Bio Labs, Bend, OR). Measurements are performed at ambient conditions on a Leica SP5 CLSM (Leica, Heidelberg, Germany) using a Leica HCX APO 20x/0.50 water immersion lens with pinhole size 6 Airy units. For imaging, a 488 nm laser at 10% power and 1% AOTF (acousto‐optic tunable filter) is used. Detection is performed with a PMT (photo‐multiplier tube) detector with gain 436 V and detection range 500–650 nm. For bleaching, 458, 476, 488, 496 and 514 nm lasers were set at 15% AOTF. Image acquisition is performed at zoom 4, yielding a field‐of‐view of 194.5 × 194.5 µm. The acquired image size is 256 × 256 pixels with pixel size 0.7598 µm stored in 16‐bit. The scan rate is 1000 Hz, yielding Δt=0.265 s. Further, we use a circular bleach region with 30 µm diameter, centred in the field of view. We use 10 prebleach frames, 4 bleach frames and 100 postbleach frames. For each of the samples, 20 replicate measurements are done in different parts of the sample.

Before analysis, the data are pre‐processed in the following manner. Firstly, the 16‐bit data are rescaled to range [0,1]. Secondly, background subtraction is performed by pixel‐wise subtraction of a Gaussian filtered (σ=5 pixels) average prebleach frame from all pre‐ and post‐bleach frames, followed by addition of the average prebleach intensity back again. The pre‐processing is the same for least squares and neural network estimation.

**FIGURE 7:**
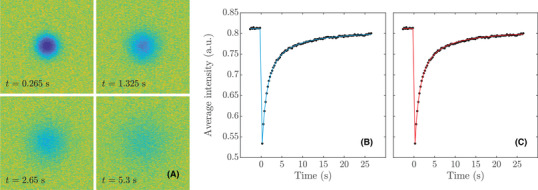
Example of a 32 w/w% sucrose data set and the corresponding fits, showing (A) the pre‐processed postbleach image data at different points in time, (B) the recovery curve (black dots) and the least squares fit (blue line) and (C) the recovery curve (black dots) and the neural network fit (red line). The two fits are plotted in different figures for clarity because they overlap almost perfectly

**FIGURE 8:**
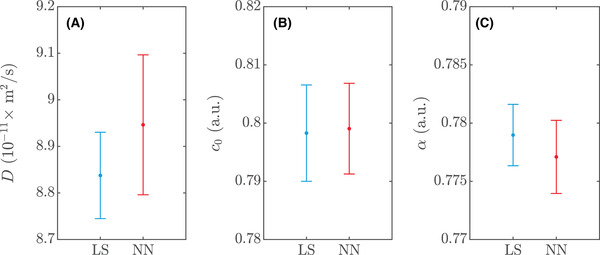
Comparison of least squares (LS) and neural network (NN) estimates for the 20 measurements performed on the 32 w/w% sucrose data sets, showing error bars with 95% confidence intervals for (A) D, (B) $c_{0}$ and (C) α

**FIGURE 9:**
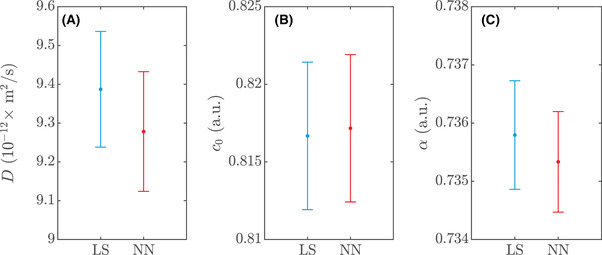
Comparison of least squares (LS) and neural network (NN) estimates for the 20 measurements performed on the 56 w/w% sucrose datasets, showing error bars with 95% confidence intervals for (A) D, (B) $c_{0}$ and (C) α

Least squares estimation is performed with 30 random initial guesses for the parameter vector for each data set to ensure convergence to the global optimum. Random guesses are uniformly distributed in the ranges $2\times 10^{-11}\leq D\leq 5\times 10^{-10}$ m${}^{2}$/s (for the 32 w/w% sucrose data sets), $2\times 10^{-12}\leq D\leq 5\times 10^{-11}$ m${}^{2}$/s (for the 56 w/w% sucrose data sets), $c_{0}$ in the intensity range of the prebleach data with a ±0.05 margin, and 0.01≤α≤1. The best fit is selected for each data set. Neural network estimation is performed in the same manner. In Figure 7, one example of a 32 w/w% sucrose dataset and the corresponding fits are shown. The estimated diffusion coefficients for the 32 w/w% sucrose datasets are (mean value and 95% confidence intervals) $8.84\times 10^{-11}$ ($\left[8.75\times 10^{-11},8.93\times 10^{-11}\right]$) m${}^{2}$/s for least squares and $8.95\times 10^{-11}$ ($\left[8.80\times 10^{-11},9.10\times 10^{-11}\right]$) m${}^{2}$/s for neural networks. The estimated diffusion coefficients for the 56 w/w% sucrose data sets are (mean value and 95% confidence intervals) $9.39\times 10^{-12}$ ($\left[9.24\times 10^{-12},9.54\times 10^{-12}\right]$) m${}^{2}$/s for least squares and $9.28\times 10^{-12}$ ($\left[9.12\times 10^{-12},9.43\times 10^{-12}\right]$) m${}^{2}$/s for neural networks. A detailed comparison of the results for least squares and neural networks is found in Figures 8 and 9 for the two measurement series. The differences are strikingly small, and indeed, a series of *t*‐tests indicates no significant difference of the means for any of the parameters or measurement series (p>0.2 in all cases). The variance is noticeably larger for the neural network estimate of D in Figure 8, and an *F*‐test indicates significant difference (p≈0.04). It is worth pointing out that for experimental data, the observed differences between the methods are not only due to how well they cope with data that satisfy the model assumptions, but also due to how they cope with the small deviations from the model assumptions that inevitably occur in real data. The values of a obtained are roughly $3\times 10^{-3}$ and hence within the range covered by the training data. As can be seen, the estimated recovery curves from least squares and the neural network coincide almost perfectly. As we pointed out earlier though, in terms of the residual sum of squares, the least squares fit is by definition optimal, and indeed, we can confirm that the neural network fits to the recovery curves are slightly worse although the difference in most cases is very small. Finally, we inspect the distribution of residuals between the fitted models and the data on the pixel level. In all cases, the residuals are almost perfectly normal distributed, verifying that the assumption of constant normal distributed noise is reasonable in this setting.

### 3.5 Computational speed

**FIGURE 10:**
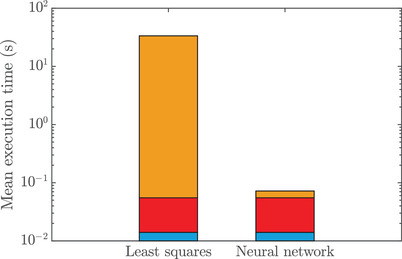
Mean execution time for least squares estimation with a single fit vs. neural network estimation, divided into preprocessing (blue), recovery curve extraction (red) and actual estimation (yellow). Note the logarithmic scale on the y axis

The computational speed of the entire workflow is not straightforward to quantify in a practical and precise way, since the least squares estimation and neural network estimation procedures are notably different. Moreover, the computations include data pre‐processing, for example, rescaling and background subtraction, followed by extraction of the recovery curve, as described in the validation on experimental data. For effective comparison, we measure the separate and cumulative execution times for pre‐processing the data by background subtraction, extracting the recovery curve and the actual estimation procedure. The data are simulated using the same parameter distribution as for the training data, after which the background is subtracted (although this is not necessary for simulated data, it is part of the workflow for experimental data) and the recovery curve is extracted. For least squares, a single fit is performed for each dataset, with initial guesses for the parameters selected randomly from the same distributions as before. As for the neural networks, the execution time of the actual prediction is measured, but not the initialization and loading of the weights since this does not have to be performed every time. Similarly, loading data and saving results are excluded from the measurement in both cases. The procedure is repeated 1,000 times, executed serially on a dual AMD Epyc 7542 with 128 threads. In Figure 10, the mean execution time for both cases are presented, split up between the three steps as described above. As can be seen, the time required for pre‐processing and recovery curve extraction is actually considerableed to the time required for the neural network estimation. For least squares estimation, in contrast, these steps are insignificant compared to the estimation. It is worth emphasizing once again that in this comparison, only a single least squares fit is performed, but in non‐linear least squares fitting it is good practice to perform multiple fits to ensure convergence to the global optimum. If instead 10 fits were performed, the difference would increase by one order of magnitude, further increasing the difference between the neural network and least squares. We stress that for the neural network, there is no counterpart to multiple fits because its output for a given set of input data is deterministic. The difference between the methods will become particularly noticeable for batch analysis of large numbers of data sets.

Implementation bias and performance differences between programming languages can of course impact the comparison. On that note, it is worth pointing out that the Matlab code relies heavily on the Optimization Toolbox and FFT transforms, both of which use very fast low‐level implementations. Long execution times for the least squares fitting is due to the complexity and detail of the model, not due to a slow implementation per se.

## 4 CONCLUSION

We have implemented a neural network approach for fast parameter estimation in FRAP, DeepFRAP, which to our knowledge is the first machine learning‐based acceleration of FRAP analysis. Using a previously developed numerical model, we generate a very large set of simulated recovery curves with realistic noise levels covering a broad range of diffusion coefficients, image intensities, amounts of bleaching and noise levels. We use the neural networks to perform non‐linear regression for parameter estimation. We implement two different neural network architectures; first, a fully connected network for estimation of all parameters jointly, and second, a set of separate fully connected networks, trained separately, for estimation of individual parameters. The latter approach is shown to give better performance for two of the three parameters; for the diffusion coefficient, the joint network gives better performance. We split and merge the networks and parts of networks that give the best performance for each parameter individually. The resulting, final neural network is comprehensively validated against least squares using both simulated and experimental data, and is found to perform very well, and be very fast compared to least squares. This implies that the neural network estimates can be used as very good initial guesses for least squares estimation in order to make the latter converge much faster than it otherwise would. The fact that the neural networks are very fast provides for obtaining, for example, diffusion coefficients as soon as possible, spending minimal time on data analysis. Indeed, recalling the three reasons mentioned in the Introduction as to why least squares can be computationally heavy (long execution time of a single fit, using multiple initial guesses to ascertain convergence, and using several measurements to assess variability), the neural network approach alleviates the first two: a single estimation is orders of magnitude faster than for least squares, and there is not even a counterpart to using an initial guess, so a single run suffices. In this fashion, the proposed method facilitates efficient use of the experimentalist's time which is the main motivation to our approach.

The neural networks could be optimized further, by exploring the hyperparameter space with respect to different architectures like fully connected networks and convolutional networks, other activation functions, dropout, batch normalization, rescaling of inputs and outputs, and using even larger datasets. Even though it remains an open question what the best choices are, the comparison between least squares and the neural network indicates that very little improvement is even possible. The training is fairly time‐consuming in the current setting, so the most interesting effort would be to shorten the training time. For this particular problem, the unusual situation arises that benchmark values for the loss are accessible by comparison to the performance of least squares estimation. Therefore, it is possible to monitor the training and stop early when an acceptable loss, relative to the least squares loss, has been reached. Indeed, in our setting, the loss with respect to the diffusion coefficient surpassed least squares after approximately 12 h of training, and this may be sufficient for practical purposes. If desired, the neural network estimate can be used as an initial guess for least squares estimation, accelerating the numerical optimization step considerably.

Although the neural network is trained on a specific set of experimental parameters, such as bleach region size, field of view and number of pre‐bleach, bleach and post‐bleach images, a similar network can be trained to cope with other parameters. The same goes for the ranges of sample parameters such as diffusion coefficients covered by the training data. In fact, if, for example, the time step Δt would change, the estimated diffusion coefficients could just be rescaled accordingly. Further, it is plausible that a neural network can perform similarly to least squares also for estimating the parameters of more complex processes than free diffusion, such as reaction‐diffusion, sub‐diffusion, and convective flow, provided that an FRAP model for that case is available such that realistic data can be simulated. Indeed, if the combination of experimental setup and FRAP model is such that least squares produces good estimation results, the same can be expected of a well‐trained neural network. It is worth pointing out though, that the attainable performance of a neural network as well as of least squares will always be limited by the complexity of the model (e.g. an increasing number of parameters is increasingly hard to estimate), noise levels, the number of collected data points, and whether the intensity inside the bleach region has fully recovered during the experiment. All in all, this is a proof‐of‐concept that can be tailored to a set of specific use‐cases in a straightforward manner, either by training from scratch or by using the network(s) already trained herein for transfer learning.

In addition, one direction for future work would be to develop a neural network model that uses the entire spatiotemporal image sequence as input. This could potentially produce better estimates in the same manner that it does so for least squares estimation. The downside would be increased complexity of the model and a significant increase in the estimation time, the computational resources required for training, and the storage space required for training data compared to a neural network trained on merely temporal data.

Finally, we make the data and code used herein publicly available^36^ to encourage and facilitate further development of machine learning‐based estimation in FRAP.
